# Quantifying Changes on OCT in Eyes Receiving Treatment for Neovascular Age-Related Macular Degeneration

**DOI:** 10.1016/j.xops.2024.100570

**Published:** 2024-06-28

**Authors:** Gabriella Moraes, Robbert Struyven, Siegfried K. Wagner, Timing Liu, David Chong, Abdallah Abbas, Reena Chopra, Praveen J. Patel, Konstantinos Balaskas, Tiarnan D.L. Keenan, Pearse A. Keane

**Affiliations:** 1 NIHR Biomedical Research Centre for Ophthalmology, Moorfields Eye Hospital NHS Foundation Trust and UCL Institute of Ophthalmology, London, United Kingdom; 2 Division of Epidemiology and Clinical Applications, National Eye Institute, National Institutes of Health, Bethesda, Maryland

**Keywords:** Antivascular endothelial growth factor, Artificial intelligence, Machine Learning, Neovascular Age-Related Macular Degeneration, Optical coherence tomography

## Abstract

**Purpose:**

Application of artificial intelligence (AI) to macular OCT scans to segment and quantify volumetric change in anatomical and pathological features during intravitreal treatment for neovascular age-related macular degeneration (AMD).

**Design:**

Retrospective analysis of OCT images from the Moorfields Eye Hospital AMD Database.

**Participants:**

A total of 2115 eyes from 1801 patients starting anti-VEGF treatment between June 1, 2012, and June 30, 2017.

**Methods:**

The Moorfields Eye Hospital neovascular AMD database was queried for first and second eyes receiving anti-VEGF treatment and had an OCT scan at baseline and 12 months. Follow-up scans were input into the AI system and volumes of OCT variables were studied at different time points and compared with baseline volume groups. Cross-sectional comparisons between time points were conducted using Mann–Whitney *U* test.

**Main Outcome Measures:**

Volume outputs of the following variables were studied: intraretinal fluid, subretinal fluid, pigment epithelial detachment (PED), subretinal hyperreflective material (SHRM), hyperreflective foci, neurosensory retina, and retinal pigment epithelium.

**Results:**

Mean volumes of analyzed features decreased significantly from baseline to both 4 and 12 months, in both first-treated and second-treated eyes. Pathological features that reflect exudation, including pure fluid components (intraretinal fluid and subretinal fluid) and those with fluid and fibrovascular tissue (PED and SHRM), displayed similar responses to treatment over 12 months. Mean PED and SHRM volumes showed less pronounced but also substantial decreases over the first 2 months, reaching a plateau postloading phase, and minimal change to 12 months. Both neurosensory retina and retinal pigment epithelium volumes showed gradual reductions over time, and were not as substantial as exudative features.

**Conclusions:**

We report the results of a quantitative analysis of change in retinal segmented features over time, enabled by an AI segmentation system. Cross-sectional analysis at multiple time points demonstrated significant associations between baseline OCT-derived segmented features and the volume of biomarkers at follow-up. Demonstrating how certain OCT biomarkers progress with treatment and the impact of pretreatment retinal morphology on different structural volumes may provide novel insights into disease mechanisms and aid the personalization of care. Data will be made public for future studies.

**Financial Disclosure(s):**

Proprietary or commercial disclosure may be found in the Footnotes and Disclosures at the end of this article.

OCT is an essential tool for the diagnosis and monitoring of retinal diseases such as neovascular age-related macular degeneration (AMD). The interpretation of OCT is reliant upon subjective assessment of anatomical and pathological structures but demonstrates low intergrader agreement and inconsistency both in clinical trials and in clinical settings.^1^, ^2^, ^3^, ^4^ Artificial intelligence (AI) approaches including deep learning offer a standardized and objective method of quantifying abnormalities and can be applied at scale to large data sets in an automated manner.^5^, ^6^, ^7^, ^8^, ^9^, ^10^

The ability to identify and quantify anatomical OCT-based ocular biomarkers may aid diagnosis, guide treatment, and provide insights into disease progression. Previous work has demonstrated the applicability of AI-derived systems to diagnose and triage major retinal diseases, including neovascular AMD,^11^ and to automatically detect and quantify retinal fluid^5^^,^^9^^,^^12^ and other clinically relevant retinal features such as hyperreflective foci (HRF), subretinal hyperreflective material (SHRM), and pigment epithelial detachment (PED).^13^ Artificial intelligence–enabled quantification has been used to improve the scientific understanding of neovascular AMD at disease presentation in first-treated and second-treated eyes^14^ and longitudinally during follow-up.^15^ By avoiding problems of low intragrader consistency and intergrader agreement, Keenan et al^10^ demonstrated substantial advantages of automated quantitative measures of retinal fluid volume using AI-acquired volumetric information when compared with manual detection of fluid in clinical and research settings.

The detection of intraretinal fluid (IRF) and subretinal fluid (SRF) has been a primary focus of AI-based quantification due to their important roles on clinical outcomes; however, anatomical features such as SHRM, PED, HRF, retinal pigment epithelium (RPE) detachment, and neurosensory retina (NSR) remain relevant in the underlying pathological processes of neovascular AMD.^16^ Accurate measurement of these various anatomical features during anti-VEGF treatment provides valuable insights into disease progression potentially leading to new quantitative management protocols. This could reduce practice variability and allow individualized pathways of care maximizing the benefits of anti-VEGF treatment in clinical practice.

In this study, we apply a validated deep learning segmentation model^11^^,^^17^ to a large clinical OCT data set of eyes undergoing anti-VEGF therapy for neovascular AMD (Moorfields Eye Hospital NHS Foundation Trust AMD Database).^18^^,^^19^ We previously reported the anatomical findings for this data set of eyes at the treatment-naive stage.^14^ In the current study, we build on this by analyzing changes in these anatomical findings longitudinally over the course of a year of anti-VEGF therapy. We make these data publicly available for replication and future investigation by the AMD research community.

## Methods

### Data Set

The Moorfields AMD data set for this study included all treatment-naive eyes that commenced anti-VEGF therapy for neovascular AMD between June 1, 2012, and June 30, 2017.^14^^,^^18^^,^^20^ Only eyes that had both an OCT scan at baseline (before the start of treatment) and at 12 months were included for analysis. Imaging data comprised 128-slice macular OCT scans (Topcon 3D OCT-2000) covering a volume of 6 × 6 × 2.3 mm. To ensure that the volumes of segmented features were reliable, images were excluded after manual review if they were of poor quality, defined as an OCT volume where the major retinal interfaces were not visible.^11^ Patient demographics included age, self-reported gender, and race/ethnicity (according to groups defined by the UK census).^21^

Imaging and clinical data were collected for the baseline visit and from 1, 2, 4, 6, and 12-month follow-up visits, in which the baseline visit marks the start of treatment, month 1 corresponds to the second loading dose injection, and month 2 refers to the third loading dose injection. Visits within 7 days of the planned time point were included (e.g., for month 1, appointments could range from 21–35 days after baseline). Although information from baseline, month 1, month 2, and month 4 was chosen to assess the response to loading doses, month 4 (also named postloading phase time point) was chosen to evaluate volumes after the 3-monthly loading doses, inclusive of visits between 105 to 119 days after baseline. Month 6 was included to assess whether an increase in fluid in month 4 regresses despite the interval remaining around 8 weeks. The final follow-up at 12 months was chosen because injection intervals are often personalized from this point onward.

All patients initiated treatment with 3 injections of either ranibizumab or aflibercept separated by 1 month between treatments. The treatment regimen, either pro re nata or “treat and extend,” was followed at the physician’s discretion.

Fellow eyes that sequentially converted to neovascular AMD and started treatment in the time period of this study were also analyzed using the same analysis strategy as first-treated eyes. All eyes were analyzed independently. If multiple scans were present on the same visit, the scan with the lowest volume of mirror and blink artifacts was selected for analysis. Where neither of these artifacts existed, the scan with the lowest volume of padding artifact, indicating less manipulation performed by the OCT device software during postprocessing and therefore, a cleaner image capture, was selected. Review and analysis of retrospective anonymized data were approved by the Moorfields Eye Hospital Institutional Review Board (ROAD17/031 and 20/HRA/2158), and the research adhered to the tenets of the Declaration of Helsinki.

### Segmentation Network

All scans were input into the previously described 3-dimensional segmentation network.^11^^,^^17^ Further details on this segmentation network have been previously published.^14^^,^^20^ The following segmented features were analyzed: IRF, SRF, PED, SHRM, NSR, RPE, and HRF. Pigment epithelial detachment was a sum of serous PED, fibrovascular PED, and drusenoid PED segmented by the network. As per the previous papers from our group, HRF was assessed as “well-circumscribed, dot- or oval-shaped lesions that are present within the intraretinal layers. They can be visualized on OCT as small lesions with equal or greater hyper-reflectivity than the RPE.”^20^

Neurosensory retina volume segmentation excluded the IRF, SRF, and SHRM components. Volumes were scaled to mm^3^ for analysis.

### Statistical Analysis

The mean and standard deviation of each of the 7 segmented features were calculated for both first-treated and second-treated eyes at each time point for which data were collected. The relative change of means was calculated as a percentage to demonstrate how the volumes changed with respect to their baseline values. The Mann–Whitney *U* test was used to assess if these relative changes were significant at each time point in both first-treated and second-treated eyes. Furthermore, the Mann–Whitney *U* test was applied to compare the relative changes between the 2 sets of eyes.

To examine biomarker volume at follow-up stratified by baseline status, first-treated eyes were divided into 2 equally sized groups, based on biomarker volume at baseline (IRF, SRF, and SHRM) being above or below the median value. Cross-sectional comparisons between these 2 groups at subsequent time points were then made. Changes in the volumes of 7 segmented features were compared between the 2 groups.

To assess whether the baseline volume of certain biomarkers (e.g., IRF) was associated with the rate of change of another biomarker volume over time (e.g., RPE), linear mixed-effects were fitted using maximum likelihood estimation with a random effect on the intercept and a cross-level interaction between time and baseline biomarker volume group. Only data for the first 12 months of the first eye were included for analysis to mitigate any bias imparted by variable practice patterns (e.g., pro re nata or treat-and-extend). Models were adjusted for age, sex, ethnicity, and visual acuity. Degrees of freedom were estimated using Satterthwaite’s approximation.

We applied a Bonferroni adjustment to the level of statistical significance to *P* < 0.00026 (193 prespecified comparisons).

All analysis was performed using Python 3.6 and R version 4.1.0 and the lme4 and lmerTest packages were used. Deidentified data for this study will be publicly available from the Dryad Digital Repository.

## Results

**Figure 1:**
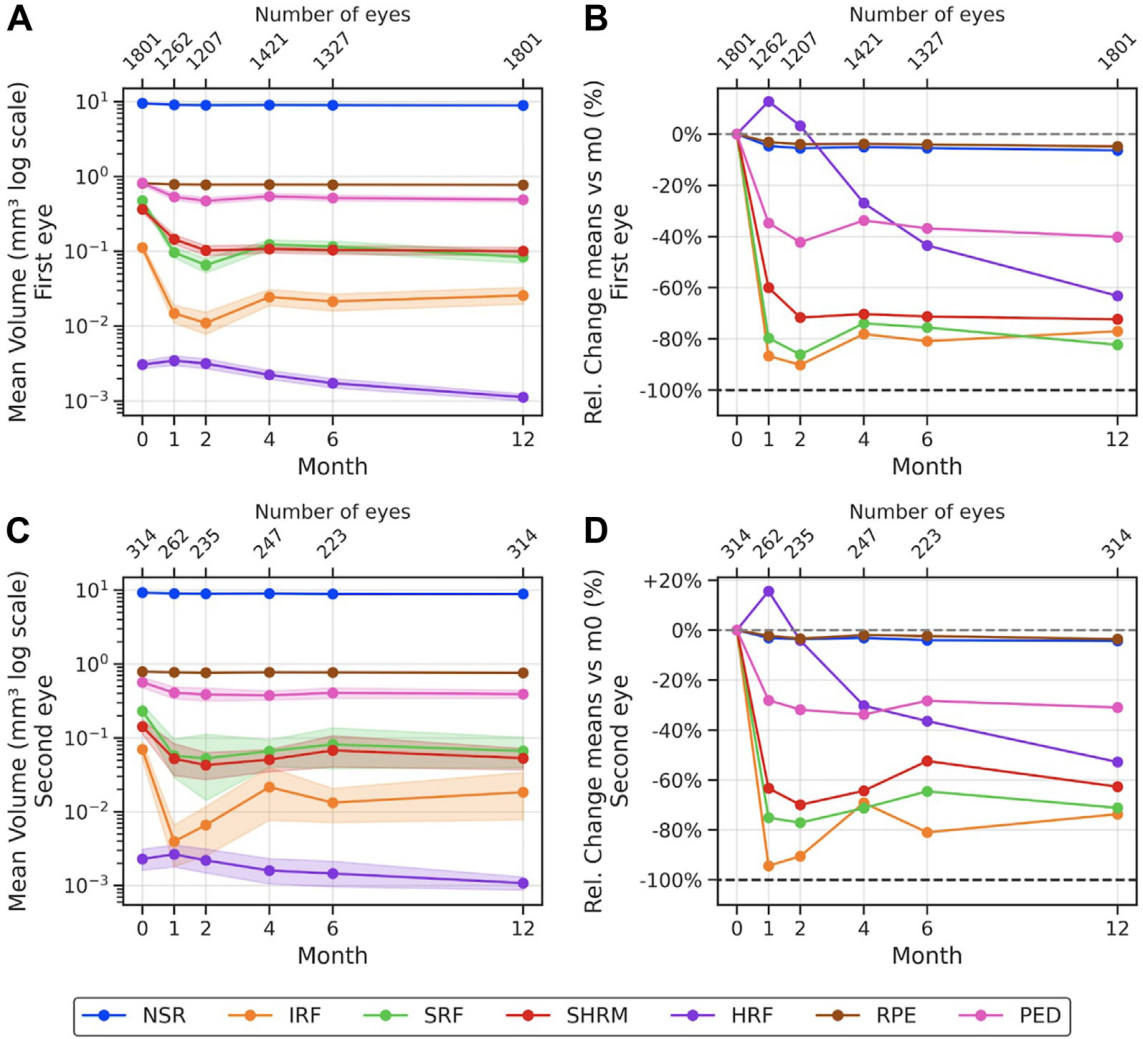
Change in mean volumes of segmented features through a 12-month period and relative change of means compared with baseline values (%), for both first-treated and second-treated eyes. **A,** Line plots illustrating absolute mean volumes of OCT segmented features in first-treated eyes throughout treatment. The volumes (mm^3^) are distributed across a logarithmic scale. Month 0 represents the first injection date. The shaded area shows the standard deviation. **B,** Line plots illustrating relative change (%) in mean volumes from month 0 of OCT segmented features in first-treated eyes. **C,** Line plots illustrating absolute mean volumes of OCT segmented features in second-treated eyes throughout treatment. The volumes (mm^3^) are distributed across a logarithmic scale. Month 0 represents the first injection date. The shaded area shows the standard deviation. **D,** Line plots illustrating relative change (%) in mean volumes from month 0 of OCT segmented features in second-treated eyes. HRF = hyperreflective foci; IRF = intraretinal fluid; NSR = neurosensory retina; PED = pigment epithelial detachment; RPE = retinal pigment epithelium; SHRM = subretinal hyperreflective material; SRF = subretinal fluid.

The data set comprised OCT scans from a total of 2115 eyes from 1801 patients, of which 1801 (85.2%) were first-treated eyes and 314 (14.8%) were second-treated eyes. The baseline demographic characteristics of the patients included in the analysis are summarized in Table 1. The mean volumes of segmented features, subdivided into first-treated and second-treated eyes, along with the mean relative change (percentage) from baseline values, are shown in Table 2 and Figure 1. Mean volumes at other time points are shown in Table S3 (available at www.ophthalmologyscience.org).

**Table 1:** Demographics of Patients Included in the Study

Demographic Characteristics		First-Treated Eye (n = 1801)	Second-Treated Eye (n = 314)
Gender	Female (%)	1089 (60.5%)	225 (71.7%)
	Male (%)	712 (39.5%)	89 (28.3%)
Race/ethnicity	White (%)	993 (55.1%)	183 (58.3%)
	Asian (%)	198 (11.0%)	24 (7.6%)
		
	Black (%)	34 (1.9%)	3 (1.0%)
	Other/Unknown (%)	576 (32.0%)	104 (33.1%)
Age (yrs)	Mean (SD)	78 (±8.6)	80 (±8.1)
Visual acuity (ETDRS letters)	Mean (SD)	55 (±15.5)	62 (±13.3)

SD = standard deviation.

**Table 2:** Mean Volumes of OCT Segmented Features in First-Treated and Second-Treated Eyes at Multiple Time Points

Segmented Feature	Month (n, Number of Eyes)	First-Treated Eye			Month (n, Number of Eyes)	Second-Treated Eye			*P* Value (Relative Change First vs Second-Treated Eye)
		Mean mm^3^ Volume (SD)	Mean % Relative Change (SD)	*P* Value (Change in Volume in First-Treated Eye versus Month 0)		Mean mm^3^ Volume (SD)	Mean % Relative Change (SD)	*P* Value (Change in Volume in Second-Treated Eye versus Month 0)	
NSR	0 (1801)	9.500 (0.942)	Reference		0 (314)	9.310 (0.802)	Reference		Reference
	4 (1421)	9.020 (0.812)	–5.06 (0.000)	**2.6 × 10**^–302^	4 (247)	9.020 (0.781)	–3.12 (0.000)	**7.7 × 10**^–52^	6.9 × 10^–4^
	12 (1801)	8.890 (0.802)	–6.36 (0.000)	**0.00**	12 (314)	8.910 (0.844)	–4.30 (0.000)	**1.7 × 10**^–62^	9.0 × 10^–4^
IRF	0 (1801)	0.112 (0.287)	Reference		0 (314)	0.070 (0.170)	Reference		Reference
	4 (1421)	0.024 (0.112)	–78.13 (0.000)	**6.6 × 10**^–166^	4 (247)	0.022 (0.126)	–69.15 (0.027)	**7.1 × 10**^–38^	0.026
	12 (1801)	0.026 (0.150)	–77.04 (0.000)	**1.4 × 10**^–181^	12 (314)	0.018 (0.002)	–73.69 (0.068)	**1.8 × 10**^–31^	0.099
SRF	0 (1801)	0.475 (0.757)	Reference		0 (314)	0.231 (0.466)	Reference		Reference
	4 (1421)	0.124 (0.335)	–73.97 (0.019)	**0.00**	4 (247)	0.066 (0.214)	–71.26 (0.001)	**5.1 × 10**^–50^	0.461
	12 (1801)	0.084 (0.320)	–82.35 (0.023)	**0.00**	12 (314)	0.067 (0.286)	–71.16 (0.030)	**3.8 × 10**^–65^	0.493
SHRM	0 (1801)	0.363 (0.642)	Reference		0 (314)	0.143 (0.284)	Reference		Reference
	4 (1421)	0.108 (0.273)	–70.33 (0.006)	**1.6 × 10**^–297^	4 (247)	0.051 (0.152)	–64.33 (0.005)	**2.2 × 10**^–37^	0.591
	12 (1801)	0.100 (0.272)	–72.37 (0.018)	**8.9 × 10**^–306^	12 (314)	0.053 (0.164)	–62.69 (0.003)	**6.2 × 10**^–49^	0.342
HRF	0 (1801)	0.003 (0.008)	Reference		0 (314)	0.002 (0.007)	Reference		Reference
	4 (1421)	0.002 (0.006)	–26.94 (0.000)	**7.0 × 10**^–50^	4 (247)	0.002 (0.005)	–30.25 (0.000)	**3.0 × 10**^–5^	0.039
	12 (1801)	0.001 (0.003)	–63.23 (0.001)	**3.5 × 10**^–118^	12 (314)	0.001 (0.002)	–52.79 (0.001)	**2.4 × 10**^–7^	3.9 × 10^–4^
RPE	0 (1801)	0.809 (0.084)	Reference		0 (314)	0.790 (0.089)	Reference		Reference
	4 (1421)	0.778 (0.094)	–3.82 (0.000)	**2.0 × 10**^–84^	4 (247)	0.774 (0.090)	–2.01 (0.000)	**2.9 × 10**^–12^	0.032
	12 (1801)	0.770 (0.096)	–4.81 (0.000)	**8.6 × 10**^–137^	12 (314)	0.761 (0.096)	–3.63 (0.000)	**1.4 × 10**^–20^	0.148
PED	0 (1801)	0.818 (1.35)	Reference		0 (314)	0.567 (0.774)	Reference		Reference
	4 (1421)	0.542 (0.899)	–33.72 (0.100)	**2.0 × 10**^–40^	4 (247)	0.376 (0.437)	–33.73 (0.002)	0.611	0.003
	12 (1801)	0.489 (0.760)	–40.20 (0.116)	**1.5 × 10**^–49^	12 (314)	0.391 (0.451)	–30.99 (0.104)	**1.7 × 10**^–4^	0.084

Mean volumes with standard deviation of segmented features in first-treated and second-treated eyes at baseline, 4, and 12 months and the mean relative change (%) from baseline values with standard deviation. Segmented voxels are converted into mm^3^. Boldface values are significant at *P* < 0.00026 after Bonferroni correction.

HRF = hyperreflective foci; IRF = intraretinal fluid; NSR = neurosensory retina; PED = pigment epithelium detachment; RPE = retinal pigment epithelium; SD = standard deviation; SHRM = subretinal hyperreflective material; SRF = subretinal fluid.

The average number of injections in the 12-month period differed depending on the drug used for treatment. Patients who underwent treatment with aflibercept only (1124) had an average of 7.34 injections, the ones receiving ranibizumab only (n = 376) had an average of 6.04 injections, and those who changed between these drugs within the 12-month period (n = 301) had an average of 8.05 injections. Subanalyses on volumetric changes subdivided by treatment drug are shown in the supplementary material (Fig S2 and Tables S4 and S5, available at www.ophthalmologyscience.org).

### Volumes during Treatment: First-Treated Versus Second-Treated Eyes

Mean volumes of all analyzed features decreased significantly from baseline to both 4 and 12 months, in both first-treated and second-treated eyes (*P* < 0.00026). For first-treated eyes, the lowest IRF and SRF volumes were observed during the loading dose phase, at month 2. A similar trend was observed in second-treated eyes, although the lowest IRF volume was at month 1. For first-treated eyes, at 12 months, mean IRF volumes slightly increased compared with month 4 values (12 months: 0.026 mm^3^ versus 4 months: 0.024 mm^3^) but were still significantly less (*P* = 1.4 × 10^–181^) than pretreatment values (baseline: 0.112 mm^3^). Second-treated eyes also had significantly less (*P* = 1.8 × 10^–31^) IRF at 12 months, compared with baseline volumes. Mean SRF volumes in second-treated eyes at 4 and 12 months were significantly less (*P* = 5.1 × 10^–50^, *P* = 3.8 × 10^–65^, respectively), compared with baseline. Additionally, mean SRF volumes at 12 months (0.067 mm^3^) had similar values to the postloading phase (0.066 mm^3^). In first-treated eyes, however, the decrease in SRF volumes at 4 and 12 months was not significant. Mean SHRM and PED volumes, in both sets of eyes, generally followed similar patterns to those of IRF/SRF, with less pronounced but substantial decreases over the first 2 months, reaching a plateau after loading phase, with minimal change to 12 months.

Retinal pigment epithelium and NSR volumes decreased by a mean (standard deviation) percentage of 4.81% (0.0) and 6.36% (0.0) in first-treated eyes, respectively, and 3.63% (0.0) and 4.30% (0.0) in second-treated eyes, from baseline to 12 months. Mean HRF volumes underwent a 26.94% and 30.25% decrease from baseline to 4 months for first-treated and second-treated eyes, respectively. In both sets of eyes, HRF volumes reached values of 0.001 mm^3^ at 12 months, significantly less (*P* = 3.5 × 10^–118^ for first-treated eyes and 3.9 × 10^–4^ for second-treated eyes) than baseline values. The relative changes in volumes from baseline to the specified time points did not show significant differences between first-treated and second-treated eyes.

### Difference in Volumes Stratified by Baseline Characteristics (First Eyes)

**Figure 3:**
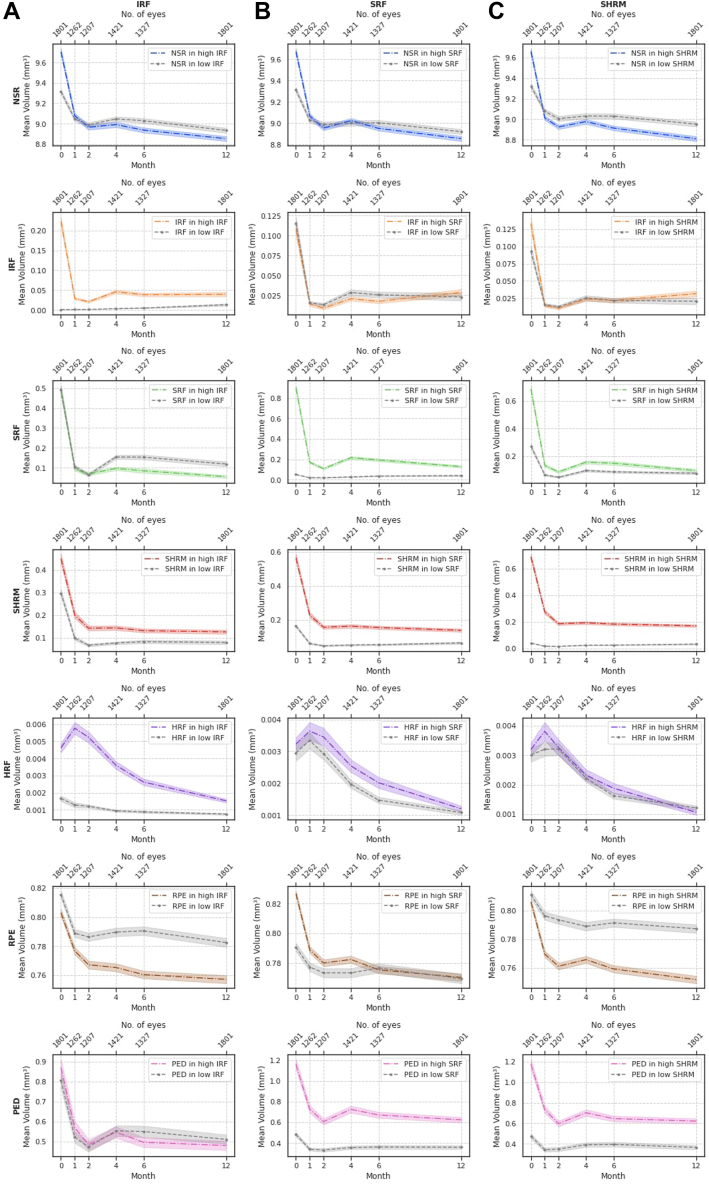
**A,** Change in volumes of each of the OCT segmented features in high and low baseline IRF volumes. **B,** Change in volumes of each of the OCT segmented features in high and low baseline SRF volumes. **C,** Change in volumes of each of the OCT segmented features in high and low baseline SHRM volumes. HRF = hyperreflective foci; IRF = intraretinal fluid; NSR = neurosensory retina; PED = pigment epithelium detachment; RPE = retinal pigment epithelium; SHRM = subretinal hyperreflective material; SRF = subretinal fluid.

First-treated eyes were divided into 2 equally sized groups, based on IRF volume at baseline being above or below the median value. Changes in the volumes of the 7 segmented features were compared between the 2 groups at multiple time points (Fig 3A, Table 6). A similar approach was used to divide eyes into groups with the following: (1) SRF volume at baseline above or below the median (Fig 3B, Table 7) and (2) SHRM volume at baseline above or below the median (Fig 3C, Table 8). This allowed us to make a descriptive observation of the changes in volumes throughout 12 months compared with baseline volume groups.

**Table 6:** Volume Comparisons at Different Time Points Stratified by Baseline Characteristics. IRF Volume Being above or below the Median Value

Segmented Features	Month 0			Month 12			Change Month 0 to Month 12		
	High IRF Group, mm^3^ (SD)	Low IRF Group, mm^3^ (SD)	*P* Value	High IRF Group, mm^3^ (SD)	Low IRF Group, mm^3^ (SD)	*P* Value	High IRF Group Month 12 to Month 0 mm^3^ (SD)	Low IRF Group Month 12 to Month 0 mm^3^ (SD)	*P* Value
NSR	9.704 (1.085)	9.286 (0.717)	**4.3 × 10**^**–21**^	8.852 (0.823)	8.933 (0.704)	0.012	–0.854 (0.971)	–0.353 (0.538)	**5.3 × 10**^**–57**^
IRF	0.223 (0.374)	0.001 (0.001)	**1.4 × 10**^**–295**^	0.040 (0.191)	0.012 (0.091)	**6.4 × 10**^**–35**^	–0.184 (0.396)	0.011 (0.091)	**6.9 × 10**^**–190**^
SRF	0.489 (0.794)	0.460 (0.718)	0.230	0.054 (0.237)	0.113 (0.383)	**1.0 × 10**^**–16**^	–0.434 (0.782)	–0.347 (0.758)	0.020
SHRM	0.450 (0.718)	0.277 (0.541)	**3.9 × 10**^**–18**^	0.127 (0.299)	0.074 (0.238)	**2.6 × 10**^**–12**^	–0.324 (0.620)	–0.202 (0.540)	**5.4 × 10**^**–13**^
HRF	0.005 (0.009)	0.002 (0.005)	**4.4 × 10**^**–41**^	0.002 (0.004)	0.001 (0.002)	**4.6 × 10**^**–16**^	–0.003 (0.008)	–0.001 (0.005)	**3.9 × 10**^**–26**^
RPE	0.803 (0.084)	0.815 (0.084)	3.1 × 10^–4^	0.757 (0.094)	0.783 (0.095)	**4.5 × 10**^**–12**^	–0.046 (0.085)	–0.032 (0.066)	**2.5 × 10**^**–5**^
PED	0.869 (1.435)	0.766 (1.252)	0.085	0.479 (0.785)	0.499 (0.735)	0.314	–0.391 (1.138)	–0.267 (0.975)	**2.8 × 10**^**–6**^

Mean volumes of first-treated eyes at baseline and at 12 months of each of the 7 segmented features (with SD) subdivided between 2 groups. Boldface values are significant at *P* < 0.00026 after Bonferroni correction.

HRF = hyperreflective foci; IRF = intraretinal fluid; NSR = neurosensory retina; PED = pigment epithelium detachment; RPE = retinal pigment epithelium; SD = standard deviation; SHRM = subretinal hyperreflective material; SRF = subretinal fluid.

**Table 7:** Volume Comparisons at Different Time Points Stratified by Baseline Characteristics. SRF Volume above or below the Median

Segmented Features	Month 0			Month 12			Change Month 0 to Month 12		
	High SRF Group, mm^3^ (SD)	Low SRF Group, mm^3^ (SD)	*P* Value	High SRF Group, mm^3^ (SD)	Low SRF Group, mm^3^ (SD)	*P* Value	High SRF Group Month 12 to Month 0 mm^3^ (SD)	Low SRF Group Month 12 to Month 0 mm^3^ (SD)	*P* Value
NSR	9.671 (0.968)	9.321 (0.882)	**2.9 × 10**^**–12**^	8.855 (0.805)	8.930 (0.798)	0.007	–0.816 (0.895)	–0.391 (0.683)	**3.8 × 10**^**–37**^
IRF	0.109 (0.311)	0.115 (0.261)	0.519	0.029 (0.142)	0.023 (0.157)	0.027	–0.080 (0.319)	–0.093 (0.286)	0.038
SRF	0.899 (0.885)	0.050 (0.056)	**1.5 × 10**^**–295**^	0.129 (0.398)	0.039 (0.207)	**2.3 × 10**^**–26**^	–0.771 (0.927)	–0.11 (0.206)	**1.8 × 10**^**–230**^
SHRM	0.566 (0.807)	0.161 (0.301)	**4.2 × 10**^**–68**^	0.138 (0.318)	0.063 (0.209)	**4.8 × 10**^**–13**^	–0.428 (0.731)	–0.098 (0.309)	**2.2 × 10**^**–53**^
HRF	0.003 (0.007)	0.003 (0.008)	**1.5 × 10**^**–8**^	0.001 (0.003)	0.001 (0.002)	0.898	–0.002 (0.006)	–0.002 (0.008)	**3.5 × 10**^**–9**^
RPE	0.827 (0.068)	0.792 (0.095)	**3.2 × 10**^**–17**^	0.771 (0.086)	0.770 (0.105)	0.325	–0.056 (0.078)	–0.022 (0.071)	**8.9 × 10**^**–23**^
PED	1.159 (1.712)	0.476 (0.686)	**1.9 × 10**^**–25**^	0.624 (0.869)	0.355 (0.603)	**1.8 × 10**^**–17**^	–0.536 (1.324)	–0.121 (0.645)	**8.3 × 10**^**–11**^

Mean volumes of first-treated eyes at baseline and at 12 months of each of the 7 segmented features (with SD) subdivided between 2 groups. Boldface values are significant at *P* < 0.00026 after Bonferroni correction.

HRF = hyperreflective foci; IRF = intraretinal fluid; NSR = neurosensory retina; PED = pigment epithelium detachment; RPE = retinal pigment epithelium; SD = standard deviation; SHRM = subretinal hyperreflective material; SRF = subretinal fluid.

**Table 8:** Volume Comparisons at Different Time Points Stratified by Baseline Characteristics. SHRM Volume above or below the Median Value

Segmented Features	Month 0			Month 12			Change Month 0 to Month 12		
	High SHRM Group, mm^3^ (SD)	Low SHRM Group, mm^3^ (SD)	*P* Value	High SHRM Group, mm^3^ (SD)	Low SHRM Group, mm^3^ (SD)	*P* Value	High SHRM Group Month 12 to Month 0 mm^3^ (SD)	Low SHRM Group Month 12 to Month 0 mm^3^ (SD)	*P* Value
NSR	9.659 (0.970)	9.333 (0.885)	**4.3 × 10**^**–12**^	8.811 (0.805)	8.974 (0.791)	**4.6 × 10**^**–7**^	–0.848 (0.905)	–0.359 (0.647)	**2.2 × 10**^**–54**^
IRF	0.134 (0.327)	0.091 (0.238)	**9.1 × 10**^**–18**^	0.031 (0.145)	0.020 (0.155)	**2.6 × 10**^**–17**^	–0.102 (0.337)	–0.071 (0.265)	6.1 × 10^–4^
SRF	0.687 (0.884)	0.262 (0.525)	**6.2 × 10**^**–71**^	0.095 (0.368)	0.073 (0.263)	**8.0 × 10**^**–5**^	–0.593 (0.908)	–0.189 (0.534)	**6.8 × 10**^**–63**^
SHRM	0.689 (0.782)	0.0380 (0.039)	**1.5 × 10**^**–295**^	0.170 (0.337)	0.031 (0.157)	**2.7 × 10**^**–87**^	–0.519 (0.726)	–0.007 (0.158)	**2.8 × 10**^**–205**^
HRF	0.003 (0.008)	0.003 (0.008)	0.077	0.001 (0.003)	0.001 (0.003)	**8.7 × 10**^**–5**^	–0.002 (0.007)	–0.002 (0.007)	**6.3 × 10**^**–6**^
RPE	0.806 (0.074)	0.812 (0.094)	0.004	0.752 (0.089)	0.789 (0.099)	**2.8 × 10**^**–19**^	–0.055 (0.079)	–0.023 (0.071)	**5.7 × 10**^**–18**^
PED	1.175 (1.666)	0.461 (0.777)	**1.3 × 10**^**–45**^	0.622 (0.827)	0.357 (0.661)	**3.0 × 10**^**–30**^	–0.553 (1.287)	–0.104 (0.706)	**1.9 × 10**^**–22**^

Mean volumes of first-treated eyes at baseline and at 12 months of each of the 7 segmented features (with SD) subdivided between 2 groups. Boldface values are significant at *P* < 0.00026 after Bonferroni correction.

HRF = hyperreflective foci; IRF = intraretinal fluid; NSR = neurosensory retina; PED = pigment epithelium detachment; RPE = retinal pigment epithelium; SD = standard deviation; SHRM = subretinal hyperreflective material; SRF = subretinal fluid.

When comparing the high baseline IRF with the low IRF group, IRF, SHRM, and HRF volumes were greater in the high IRF group at all subsequent time points. Interestingly, RPE volumes were higher in the low IRF group compared with the high IRF group throughout the follow-up period. The volumes of all 7 segmented features decreased from baseline to 12 months in both high and low IRF groups, apart from IRF volume in the low IRF group (which increased 0.011 mm^3^ after 1 year). The greatest absolute decreases were seen in NSR volume (–0.854 mm^3^), followed by SRF volume (–0.434 mm^3^), both in the high IRF group.

Additionally, when comparing the high baseline SRF versus the low SRF group, the segmented features that presented greater volumes in the high SRF group at all subsequent time points were SRF, SHRM, HRF, RPE, and PED. Eyes in the low SRF group tended to maintain SRF volumes close to 0 throughout the entire follow-up period. Intraretinal fluid volumes at baseline and at 12 months had no significant differences between the high and low SRF groups. The SHRM volume in both high IRF and high SRF groups showed a very similar curve from baseline to 12 months, decreasing by 0.324 mm^3^ and 0.428 mm^3^, respectively.

Eyes in the high baseline SHRM group tended to start with higher NSR volumes, although by 12 months these eyes presented lower NSR volumes compared with the low baseline SHRM group. A similar pattern was seen for RPE where, by the end of the first year, eyes with high baseline SHRM volumes presented significantly lower RPE volumes. The SHRM volume in both high baseline IRF and high baseline SRF groups showed very similar curves from baseline to 12 months, decreasing by 0.324 mm^3^ and 0.428 mm^3^, respectively.

To provide additional visualization of the data, the first-treated eyes were divided into the top and bottom 5% of IRF, SRF, and SHRM volumes, and changes in volumes of the segmented features were compared between the 2 groups at multiple time points (Fig S4A–C, available at www.ophthalmologyscience.org, for IRF, SRF, and SHRM accordingly). Trends in volumes show similar patterns as those observed in Figure 3A–C.

### Rates of Biomarker Change Stratified by Baseline Characteristics (First Eyes)

Rates of biomarker change were then investigated between groups stratified by baseline biomarker volume (Tables S9–S13, available at www.ophthalmologyscience.org). Those who presented with higher SRF, IRF, and SHRM at baseline had faster reductions in RPE volume over time compared with lower groups. Those with higher IRF volume at baseline also had a significantly faster reduction in NSR volume but not SHRM compared with the lower IRF group. Higher RPE volume at baseline was associated with a slower reduction in SRF volume over time.

## Discussion

The use of AI systems to facilitate quantitative analyses of an increasing stream of imaging data, particularly from OCT, has been studied in the ophthalmic community.^9^^,^^10^^,^^12^^,^^14^^,^^22^ Currently, disease activity and retreatment decisions are determined using an assessment of central subfield thickness and the qualitative presence of fluid, in both clinical trials and real-world settings.^23^, ^24^, ^25^, ^26^ However, several studies have demonstrated that discriminating between fluid types is important because of their different effects on visual function.^12^^,^^27^^,^^28^ Early fluid detection and precise volume calculations aim to help retreatment decisions and personalize management protocols. Through the application of a deep learning-based segmentation algorithm to OCT scans from the Moorfields AMD Database, we analyzed a range of segmented features of patients undergoing anti-VEGF treatment for neovascular AMD throughout 12 months. Along with IRF, SRF, and PED volumes, additional segmented features including NSR, RPE, HRF, and SHRM were evaluated over time.

Pathological features that reflect exudation, including pure fluid components (IRF and SRF) and those with a mixture of fluid and fibrovascular tissue (PED and SHRM), displayed relatively similar trends in response to anti-VEGF treatment over the 12-month period. Keenan et al^10^ analyzed data sets from diverse clinical settings and observed that IRF and SRF volumes decreased rapidly from baseline to subsequent visits. Our data confirm these trends, consistent with previous reports, showing the greatest decreases in IRF and SRF from the start of treatment to after the first injection,^15^^,^^22^^,^^29^ after which volumes remain relatively low up to 1 year.^9^^,^^25^ We observed that IRF volumes decreased by a greater extent and at a faster rate than SRF, which corroborates the responsiveness of IRF to anti-VEGF therapy shown in previous studies.^22^^,^^30^ Mean SRF volumes decreased significantly in both high and low baseline IRF groups. However, after the loading doses and beyond, the mean SRF was consistently higher in the low baseline IRF group. One could argue that in the high baseline IRF group, the treatment regimen was likely more aggressive and that in the low baseline IRF group, there may be eyes with small SRF volumes that were either being tolerated^28^ or persisted despite injections.^31^

Although SHRM and PED volumes also respond rapidly to anti-VEGF drugs, they demonstrate a less pronounced and slower reduction over time. This is hypothesized to be related to their mixed anatomical composition of serum, fibrin, and inflammatory cells^32^—although the active macular neovascularization (MNV) fluid component rapidly decreases with the anti-VEGF effect, the nonresponsive fibrotic portion persists.^9^^,^^33^, ^34^, ^35^ Longitudinal analysis showed that significantly greater SHRM volumes were seen in the high baseline IRF and SRF volume groups throughout the 1-year follow-up. This suggests that, in the group with high IRF volume at baseline, we have advanced presentations of neovascular AMD, where we expect more fibrotic material associated with SHRM. Additionally, eyes with higher SHRM at baseline demonstrate a more delayed resolution of SHRM volumes over time. This corroborates recent studies suggesting that SHRM itself may be separately considered a biomarker in neovascular AMD, where eyes with greater SHRM at a treatment-naive state are at a higher risk of fibrosis.^36^^,^^37^ The delayed reduction of SHRM could be explained because the greater SHRM at baseline could be either fibrosis or scarring. Both IRF and SRF had significantly greater volumes at baseline in the high baseline SHRM group compared with the low baseline SHRM group. In both groups, the volumes of IRF and SRF had an expected decrease during the anti-VEGF loading dose period. By 12 months, although the difference in volumes of IRF and SRF among the SHRM groups is less significant, the baseline high SHRM group still shows higher values of both IRF and SRF, corroborating the relationship of higher SHRM volumes at the start of treatment with poor prognosis.

Both NSR and RPE showed similar gradual reductions in volume over time, which were more modest than those seen for exudative features. This may reflect neurodegenerative changes over time owing to either neovascular or nonneovascular AMD. The decrease of NSR and RPE may represent neurodegeneration associated with complete or incomplete RPE and outer retinal atrophy, where there is a loss of ellipsoid and interdigitation zones associated with thinning of the outer nuclear layer.^32^^,^^38^ This may suggest that inner and outer retinal degeneration occur concurrently. Additionally, this could represent a difference in the way the model segments RPE in those with fluid, i.e., RPE is measured as having more volume over PEDs, and thus reducing its volume as PEDs flatten out with treatment. The reduction in NSR could also represent a reduction in noncystic thickening due to the effect of anti-VEGF. Longitudinal analyses showed that both NSR and RPE volumes had a greater decrease in the high baseline IRF group. One hypothesis is that eyes in the high IRF group showed a rapid initial decline in NSR volume due to anti-VEGF effect on the thickening from noncystic IRF, whereas after the loading phase, both groups suffered similar rates of retinal atrophy in response to the ongoing IRF volume at those subsequent time points. Possible explanations involve IRF being associated with a higher risk of RPE atrophy and the increased presence of more advanced/delayed type 1 and 2 MNV along with type 3 MNV lesions in the IRF high-volume group. Studies suggest that type 3 MNV lesions have a high rate of progression to complete RPE and outer retinal atrophy over time, partially because of to its prevalence in older patients and with AMD-specific genotypes, which already increases the risk of atrophy.^39^, ^40^, ^41^, ^42^ Additionally, type 3 MNVs are usually preceded by the migration of RPE cells from the monolayer into the NSR, which may leave a gap in the RPE layer.^41^ In a study by the CATT group, IRF presence at baseline was associated with double the risk of GA development.^40^ This correlates with a previous discussion by our group and another report,^14^^,^^40^ where IRF has a negative correlation with VA. Furthermore, because greater PED volumes are present in the high IRF group at baseline (although not significantly different from the low IRF group), one could argue that the greater reduction in RPE volume from the high IRF group is pathophysiologically involved with the resolution of PEDs with treatment which contributes to RPE atrophy.

On the other hand, eyes with higher SRF volumes at baseline had higher RPE volumes throughout the first year of treatment. The group with high SRF at baseline presumably contained a large proportion of eyes with MNV type 1 lesions (typically considered less aggressive in their clinical course) and fewer MNV type 3 lesions (where we might expect more RPE atrophy over time). Reports from the HARBOR, CATT, and IVAN trials included the presence at baseline of intraretinal cysts in the study eye as a risk factor for atrophy development, whereas the presence of baseline SRF was associated with a lower rate of atrophy development.^41^^,^^43^^,^^44^ In particular, in a post hoc analysis of the HARBOR trial, both increased SRF volume at baseline and treatment-resistant SRF were associated with a lower risk of macular atrophy.^45^ It has been suggested that SRF may be an indicator of a persistent MNV type 1 lesion, where the neovascular net and functional choriocapillaris layers offer a nourishing environment for RPE and thus protect against the development of atrophy.^41^^,^^46^^,^^47^ Siedlecki et al^48^ analyzed eyes with neovascular AMD and an SRF-only phenotype and reported a low incidence of macular atrophy in their study, with 22.4% at year 5, suggesting that SRF might indeed act as a buffer against the direct toxic effects of the MNV or contain neuroprotective factors promoting RPE and outer retinal survival. However, as shown in the IRF groups, eyes with higher SRF at baseline had a more rapid reduction in RPE volume over the first 12 months (perhaps due to the fact that the SRF protective factor to the RPE atrophy is impacted by the remaining SRF volumes throughout treatment, which are very different from baseline volumes).

Eyes in the high baseline IRF group showed higher volumes of HRF than those in the low IRF volume group at the same time period. After 1 year of anti-VEGF therapy, although HRF in both groups decreased substantially, eyes in the high IRF group still showed significantly higher HRF volumes. Hyperreflective foci were first reported by Coscas et al^49^ and since then, several possible theories for their origins have been proposed. One hypothesis is that they appear as a result of vascular hyperpermeability and disruption of the blood–retinal barrier.^50^ Studies in patients with both neovascular AMD and diabetic macular edema show that HRF decrease with anti-VEGF therapy.^34^^,^^50^, ^51^, ^52^, ^53^ In the report by Segal et al,^50^ the amount of HRF was significantly reduced after treatment at each follow-up visit, when compared with baseline. This was supported by a previous study,^54^ which reported a 54% reduction of HRF in neovascular AMD eyes after 3 monthly injections of anti-VEGF. In our study, HRF volumes reached almost 0 by 12 months, with a decrease of 63% from baseline values. This trend emphasizes the strong correlation between IRF and HRF observed at baseline.^14^

Our comparison between first-treated and second-treated eyes over time revealed very similar patterns between the 2 sets of eyes. As previously described by our group,^14^ second-treated eyes tend to present lower IRF and SRF volumes at baseline, likely due to earlier diagnosis. As seen in Table 2, first-treated eyes presented an absolute greater change in volumes of all 7 segmented features in response to anti-VEGF treatment from baseline to 12 months, which again reinforces the argument that first-treated eyes tend to present at a later/more advanced stage of the disease.

The limitations of this study include its retrospective nature and the variability of anti-VEGF drugs and treatment protocols used. At present, the AI system is also unable to present information regarding the location of the features within the OCT volume scan and to distinguish between subtypes of visually similar features. As a descriptive report, our aim was to foment discussions around volumetric calculations that could provide new insights into the pathophysiology of the disease. We present a study with a large number of patients in clinical setting conditions reflecting routine practice. We believe that AI will have great value at an individual patient level to quantify change in volumes in an automated way and personalize treatment. By making the data openly available, we strongly believe these data will help the clinical community to compare studies and encourage novel approaches to the data. For clinical trials, the demonstration of how certain OCT biomarkers progress with treatment may provide novel insights into disease mechanisms, therapeutic pharmacodynamics, and pharmacokinetics.
