# Supplementary material for: Quantifying Changes on OCT in Eyes Receiving Treatment for Neovascular Age-Related Macular Degeneration
Source: Ophthalmol Sci. 2024 Jun 28;4(6):100570. doi: 10.1016/j.xops.2024.100570 (PMC11367487; doi:10.1016/j.xops.2024.100570)
Supplement: Supplementary Figure S2 [file mmc1.pdf]

Supplementary Figure S2:
Change in mean volumes of segmented features through a 12-month period and relative change of means compared to baseline values (%), for both first and second-treated eyes, receiving intravitreal injections of either Ranibizumab-only or Aflibercept-only.

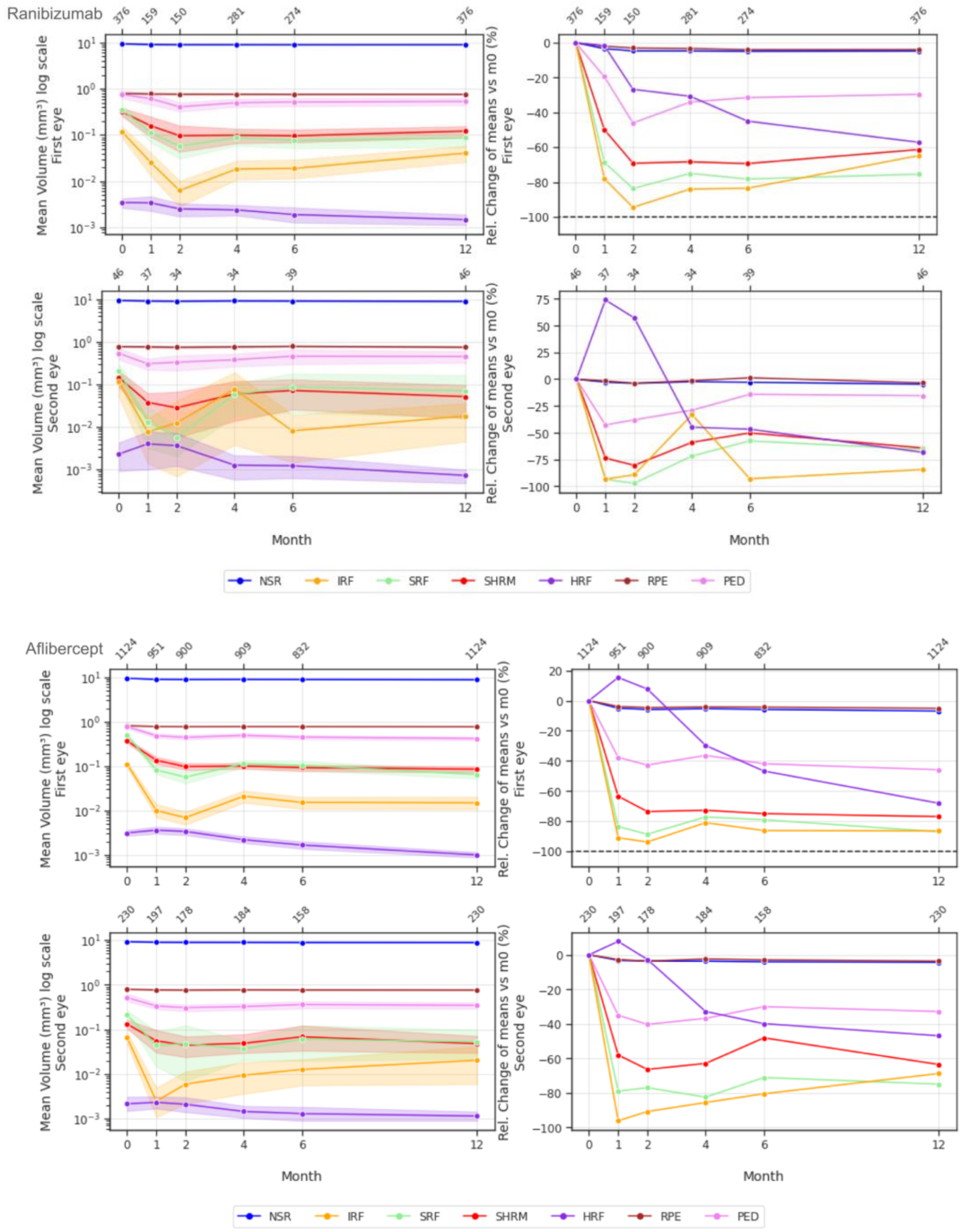

Figure S2: Change in mean volumes of segmented features through a 12-month period and relative change of means compared to baseline values (%), for both first and second-treated eyes, receiving intravitreal injections of either Ranibizumab-only or Aflibercept-only. Changes in volumes of NSR, IRF, SRF, SHRM and HRF from baseline to 12 months were significantly different ($p<0.002$, Bonferroni-corrected) in first treated eyes that received Ranibizumab-only versus Aflibercept-only. NSR = neurosensory retina, RPE = retinal pigment epithelium, IRF = intraretinal fluid, SRF = subretinal fluid, SHRM = subretinal hyperreflective material, HRF = hyperreflective foci, PED = pigment epithelium detachment.
