# Supplementary material for: Quantifying Changes on OCT in Eyes Receiving Treatment for Neovascular Age-Related Macular Degeneration
Source: Ophthalmol Sci. 2024 Jun 28;4(6):100570. doi: 10.1016/j.xops.2024.100570 (PMC11367487; doi:10.1016/j.xops.2024.100570)
Supplement: Supplementary Figure S10 [file mmc2.pdf]

## Supplementary Table S10

### Association between retinal biomarkers at follow up and secondary exposure variables stratified by NSR baseline volume (first treated eyes)

| Characteristic | | IRF volume1 | | SRF volume1 | | NSR volume | | RPE volume | | SHRM volume1 | |
|---|---|---|---|---|---|---|---|---|---|---|---|
| | | **β (95% CI)** | ***p value*** | **β (95% CI)** | ***p value*** | **β (95% CI)** | ***p value*** | **β (95% CI)** | ***p value*** | **β (95% CI)** | ***p value*** |
| **Age** | Per decile | 0.23 (-0.04, 0.50) | 0.09 | -0.83 (-1.06, -0.60) | **$1.3 \times 10^{-12}$** | -0.14 (-0.17, -0.10) | **$3.2 \times 10^{-13}$** | -0.25 (-0.30, -0.20 | **$1.0 \times 10^{-16}$** | -0.07 (-0.34, 0.20) | 0.6 |
| **Sex** | Female | **Reference** | | **Reference** | | **Reference** | | **Reference** | | **Reference** | |
| | Male | 0.26 (-0.21, 0.72) | 0.28 | -0.03 (-0.42, 0.36) | 0.87 | 0.10 (0.04, 0.16) | $2.4 \times 10^{-3}$ | 0.00 (-0.08, 0.09) | 0.91 | -0.11 (-0.57, 0.35) | 0.63 |
| **Ethnicity** | White | **Reference** | | **Reference** | | **Reference** | | **Reference** | | **Reference** | |
| | Asian | -0.48 (-0.99, 0.03) | 0.06 | -0.18 (-0.61, 0.25) | 0.41 | -0.10 (-0.17, -0.03) | $4.3 \times 10^{-3}$ | -0.00 (-0.09, 0.09) | 1 | 0.40 (-0.10, 0.90) | 0.12 |
| | Black | 2.49 (0.85, 4.13) | $3.0 \times 10^{-3}$ | 0.43 (-0.96, 1.82) | 0.55 | -0.20 (-0.43, 0.03) | 0.08 | 0.45 (0.16, 0.74) | $2.5 \times 10^{-3}$ | 0.98 (-0.65, 2.61) | 0.24 |
| | Other | -0.07 (-0.84, 0.70) | 0.86 | -0.39 (-1.05, 0.26) | 0.24 | -0.19 (-0.30, -0.09) | $3.5 \times 10^{-4}$ | 0.00 (-0.13, 0.14) | 0.97 | 0.68 (-0.09, 1.44) | 0.08 |
| **Time** | Per month | -0.29 (-0.35, -0.24) | **$1.0 \times 10^{-16}$** | -0.34 (-0.39, -0.30) | **$1.0 \times 10^{-16}$** | -0.03 (-0.03, -0.02) | **$1.0 \times 10^{-16}$** | -0.03 (-0.03, -0.02) | **$1.0 \times 10^{-16}$** | -0.27 (-0.32, -0.23) | **$1.0 \times 10^{-16}$** |
| **Visual acuity** | Per letter | -0.07 (-0.09, -0.06) | **$1.0 \times 10^{-16}$** | -0.02 (-0.04, -0.01) | $2.4 \times 10^{-4}$ | 0.00 (0.00, 0.01) | **$2.6 \times 10^{-6}$** | 0.01 (0.01, 0.02) | **$1.0 \times 10^{-16}$** | -0.08 (-0.10, -0.07) | **$1.0 \times 10^{-16}$** |
| **Baseline NSR volume** | Low | **Reference** | | **Reference** | | **Reference** | | **Reference** | | **Reference** | |
| | High | 2.58 (2.02, 3.14) | **$1 \times 10^{-16}$** | 0.80 (0.33, 1.27) | $8.8 \times 10^{-4}$ | 1.40 (1.33,1.47) | **$1.0 \times 10^{-16}$** | 0.54 (0.46, 0.63) | **$1.0 \times 10^{-16}$** | 0.75 (0.23, 1.27) | $5.1 \times 10^{-3}$ |
| **Time: high NSR** | Interaction | 0.00 (-0.07, 0.07) | 0.98 | -0.03 (-0.09, 0.03) | 0.31 | -0.05 (-0.06, -0.04) | **$1.0 \times 10^{-16}$** | -0.01 (-0.01, 0.00) | 0.09 | 0.01 (-0.05, 0.07) | 0.68 |

**Supplementary Table S10:** Association between retinal biomarkers at follow up and secondary exposure variables stratified by NSR baseline volume. Note that the interaction effect indicates rate of biomarker change. Bolded values were significant at P<0.00026 after Bonferroni correction. NSR = neurosensory retina, RPE = retinal pigment epithelium, IRF = intraretinal fluid, SRF = subretinal fluid, PED = pigment epithelium detachment, SHRM = subretinal hyperreflective material, HRF = hyperreflective foci, CI= Confidence interval.
