# Supplementary material for: Quantifying Changes on OCT in Eyes Receiving Treatment for Neovascular Age-Related Macular Degeneration
Source: Ophthalmol Sci. 2024 Jun 28;4(6):100570. doi: 10.1016/j.xops.2024.100570 (PMC11367487; doi:10.1016/j.xops.2024.100570)
Supplement: Supplementary Figure S11 [file mmc3.pdf]

Supplementary Table S11

Association between retinal biomarkers at follow up and secondary exposure variables stratified by RPE baseline volume (first-treated eyes)

Characteristic		IRF volume1		SRF volume1		NSR volume		RPE volume		SHRM volume1	
		**β (95% CI)**	***p value***	**β (95% CI)**	***p value***	**β (95% CI)**	***p value***	**β (95% CI)**	***p value***	**β (95% CI)**	***p value***
**Age**	Per decile	0.13 (-0.15, 0.42)	0.36	-0.75 (-0.98, -0.52)	**2.9×10^{-10}**	-0.16 (-0.21, -0.11)	**1.4×10^{-10}**	-0.12 (-0.20, -0.12)	**5.7×10^{-14}**	-0.17 (-0.44, 0.10)	0.23
**Sex**	Female	**Reference**		**Reference**		**Reference**		**Reference**		**Reference**	
	Male	0.18 (-0.30, 0.66)	0.46	-0.03 (-0.43, 0.36)	0.86	0.07 (-0.01, 0.15)	0.11	0.01 (-0.06, 0.08)	0.84	-0.14 (-0.60, 0.32)	0.54
**Ethnicity**	White	**Reference**		**Reference**		**Reference**		**Reference**		**Reference**	
	Asian	-0.67 (-1.19, -0.14)	0.01	-0.24 (-0.66, 0.19)	0.27	-0.19 (-0.29, -0.10)	**2.8×10^{-5}**	-0.05 (-0.13, 0.02)	0.17	0.35 (-0.16, 0.85)	0.18
	Black	1.81 (0.11, 3.51)	0.04	0.13 (-1.25, 1.51)	0.85	-0.54 (-0.84, -0.24)	3.9×10^{-4}	0.17 (-0.07, 0.42)	0.17	0.84 (-0.79, 2.48)	0.31
	Other	-0.12 (-0.93, 0.68)	0.76	-0.46 (-1.12, 0.19)	0.16	-0.24 (-0.38, -0.10)	9.7×10^{-4}	-0.08 (-0.19, 0.04)	0.2	0.69 (-0.08, 1.47)	0.08
**Time**	Per month	-0.34 (-0.39, -0.28)	**1.0×10^{-16}**	-0.42 (-0.46, -0.37)	**1.0×10^{-16}**	-0.05 (-0.06, -0.04)	**1.0×10^{-16}**	-0.02 (-0.03, -0.02)	**7.1×10^{-16}**	-0.27 (-0.31, -0.23)	**1.0×10^{-16}**
**Visual acuity**	Per letter	-0.08 (-0.10, -0.07)	**1.0×10^{-16}**	-0.03 (-0.04, -0.02)	**5.4×10^{-6}**	0.00 (0.00, 0.00)	0.86	0.01 (0.01, 0.01)	**4.2×10^{-12}**	-0.08 (-0.10, -0.07)	**1.0×10^{-16}**
**Baseline RPE volume**	Low	**Reference**		**Reference**		**Reference**		**Reference**		**Reference**	
	High	0.31 (-0.27, 0.90)	0.3	0.54 (0.06, 1.01)	0.03	0.53 (0.44, 0.62)	**1×10^{-16}**	1.15 (1.08, 1.23)	**1.0×10^{-16}**	-0.32 (-0.85, 0.22)	0.24
**Time: high RPE**	Interaction	0.09 (0.01, 0.16)	0.02	0.11 (0.05, 0.17)	4.3×10^{-4}	-0.01 (-0.06, 0.00)	0.07	-0.02 (-0.03, -0.02)	**9.2×10^{-10}**	0.01 (-0.05, 0.07	0.84

Supplementary Table S11: Association between retinal biomarkers at follow up and secondary exposure variables stratified by RPE baseline volume. Note that the interaction effect indicates rate of biomarker change. Bolded values were significant at P<0.00026 after Bonferroni correction. NSR = neurosensory retina, RPE = retinal pigment epithelium, IRF = intraretinal fluid, SRF = subretinal fluid, PED = pigment epithelium detachment, SHRM = subretinal hyperreflective material, HRF = hyperreflective foci, CI= Confidence interval.
