# Supplementary material for: Quantifying Changes on OCT in Eyes Receiving Treatment for Neovascular Age-Related Macular Degeneration
Source: Ophthalmol Sci. 2024 Jun 28;4(6):100570. doi: 10.1016/j.xops.2024.100570 (PMC11367487; doi:10.1016/j.xops.2024.100570)
Supplement: Supplementary Table S3 [file mmc4.pdf]

Supplementary Table S3

Mean volumes of OCT segmented features in first and second-treated eyes at multiple time-points up to 48 months

Segmented Feature	Month (n number eyes)	First treated eye			Month (n number eyes)	Second treated eye			P-value (rel. change first vs second-treated eye)
		Mean mm3 volume (SD)	Mean% relative change (SD)	P-value (change in volume in first-treated eye versus m0)		Mean mm3 volume (SD)	Mean% relative change (SD)	P-value (change in volume in second-treated eye versus m0)	
**NSR**	0 (1801)	9.500 (0.942)	Reference		0 (314)	9.310 (0.802)	Reference		Reference
	1 (1262)	9.060 (0.771)	-4.64 (0.000)	0.00	1 (262)	9.020 (0.752)	-3.13 (0.000)	1.4×10^{-61}	0.010
	4 (1421)	9.020 (0.812)	-5.06 (0.000)	2.6×10^{-302}	4 (247)	9.020 (0.781)	-3.12 (0.000)	7.7×10^{-52}	0.001
	6 (1327)	8.980 (0.791)	-5.43 (0.000)	0.00	6 (223)	8.930 (0.982)	-4.05 (0.000)	1.2×10^{-58}	0.000
	12 (1801)	8.890 (0.802)	-6.36 (0.000)	0.00	12 (314)	8.910 (0.844)	-4.30 (0.000)	1.7×10^{-62}	0.001
	24 (1062)	8.800 (0.913)	-7.33 (0.000)	0.00	24 (180)	8.790 (0.711)	-5.57 (0.000)	4.8×10^{-51}	0.051
	48 (539)	8.720 (0.795)	-8.15 (0.000)	0.00	48 (66)	8.610 (0.890)	-7.49 (0.000)	7.9×10^{-74}	0.391
**IRF**	0 (1801)	0.112 (0.287)	Reference		0 (314)	0.070 (0.170)	Reference		Reference
	1 (1262)	0.015 (0.075)	-86.70 (0.005)	1.9×10^{-219}	1 (262)	0.004 (0.020)	-94.40 (0.009)	1.3×10^{-54}	0.000
	4 (1421)	0.024 (0.112)	-78.10 (0.064)	6.6×10^{-166}	4 (247)	0.022 (0.126)	-69.20 (0.027)	7.1×10^{-38}	0.025
	6 (1327)	0.021 (0.106)	-80.90 (0.009)	5.2×10^{-170}	6 (223)	0.013 (0.054)	-81.00 (0.450)	4.0×10^{-28}	0.106
	12 (1801)	0.026 (0.150)	-77.00 (0.132)	1.4×10^{-181}	12 (314)	0.018 (0.123)	-73.70 (0.068)	1.8×10^{-31}	0.099
	24 (1062)	0.029 (0.168)	-74.10 (0.084)	1.9×10^{-129}	24 (180)	0.016 (0.131)	-76.50 (0.038)	1.1×10^{-28}	0.113
	48 (539)	0.028 (0.134)	-74.60 (1.300)	2.5×10^{-113}	48 (66)	0.009 (0.035)	-87.30 (0.015)	1.9×10^{-19}	0.340
**SRF**	0 (1801)	0.475 (0.757)	Reference		0 (314)	0.231 (0.466)	Reference		Reference
	1 (1262)	0.096 (0.296)	-79.70 (0.001)	0.00	1 (262)	0.057 (0.280)	-75.10 (0.001)	9.5×10^{-69}	0.010
	4 (1421)	0.124 (0.335)	-74.00 (0.019)	0.00	4 (247)	0.066 (0.214)	-71.30 (0.001)	5.1×10^{-50}	0.461
	6 (1327)	0.116 (0.366)	-75.60 (0.006)	4.5×10^{-300}	6 (223)	0.082 (0.375)	-64.50 (0.008)	8.7×10^{-51}	0.548
	12 (1801)	0.084 (0.320)	-82.40 (0.023)	0.00	12 (314)	0.067 (0.286)	-71.20 (0.030)	3.8×10^{-65}	0.493
	24 (1062)	0.053 (0.172)	-88.90 (0.114)	0.00	24 (180)	0.060 (0.177)	-74.00 (1.160)	5.3×10^{-45}	0.810
	48 (539)	0.045 (0.184)	-90.40 (0.413)	1.9×10^{-298}	48 (66)	0.020 (0.046)	-91.50 (0.003)	2.2×10^{-44}	0.418
**SHRM**	0 (1801)	0.363 (0.642)	Reference		0 (314)	0.143 (0.284)	Reference		Reference
	1 (1262)	0.145 (0.402)	-60.00 (0.014)	0.00	1 (262)	0.052 (0.214)	-63.30 (0.000)	1.1×10^{-59}	0.011
	4 (1421)	0.108 (0.273)	-70.30 (0.006)	1.6×10^{-297}	4 (247)	0.051 (0.152)	-64.30 (0.005)	2.2×10^{-37}	0.591

	6 (1327)	0.104 (0.263)	-71.30 (0.014)	3.0×10^{-302}	6 (223)	0.068 (0.270)	-52.40 (0.005)	3.8×10^{-34}	0.821
	12 (1801)	0.100 (0.272)	-72.40 (0.018)	8.9×10^{-306}	12 (314)	0.053 (0.164)	-62.70 (0.003)	6.2×10^{-49}	0.342
	24 (1062)	0.094 (0.255)	-74.20 (0.042)	1.8×10^{-272}	24 (180)	0.056 (0.152)	-60.60 (0.010)	4.5×10^{-34}	0.605
	48 (539)	0.098 (0.224)	-73.00 (0.044)	5.8×10^{-200}	48 (66)	0.099 (0.210)	-30.30 (0.038)	8.2×10^{-30}	0.742
**HRF**	0 (1801)	0.003 (0.008)	Reference		0 (314)	0.002 (0.007)	Reference		Reference
	1 (1262)	0.003 (0.009)	12.80 (0.001)	1.2×10^{-9}	1 (262)	0.003 (0.007)	15.60 (0.000)	4.7×10^{-2}	0.109
	4 (1421)	0.002 (0.006)	-26.90 (0.000)	7.0×10^{-50}	4 (247)	0.002 (0.005)	-30.20 (0.000)	3.0×10^{-5}	0.039
	6 (1327)	0.002 (0.005)	-43.50 (0.001)	2.3×10^{-68}	6 (223)	0.001 (0.004)	-36.40 (0.001)	3.2×10^{-11}	0.445
	12 (1801)	0.001 (0.003)	-63.20 (0.001)	3.5×10^{-118}	12 (314)	0.001 (0.002)	-52.80 (0.001)	2.4×10^{-7}	0.000
	24 (1062)	0.001 (0.002)	-73.40 (0.001)	4.6×10^{-103}	24 (180)	0.001 (0.002)	-63.80 (0.001)	1.8×10^{-12}	0.023
	48 (539)	0.001 (0.004)	-62.60 (0.004)	1.8×10^{-69}	48 (66)	0.001 (0.002)	-57.00 (0.001)	4.1×10^{-5}	0.093
**RPE**	0 (1801)	0.809 (0.084)	Reference		0 (314)	0.790 (0.089)	Reference		Reference
	1 (1262)	0.784 (0.086)	-3.14 (0.000)	4.6×10^{-125}	1 (262)	0.772 (0.091)	-2.26 (0.000)	4.4×10^{-13}	0.002
	4 (1421)	0.778 (0.094)	-3.82 (0.000)	2.0×10^{-84}	4 (247)	0.774 (0.090)	-2.01 (0.000)	2.9×10^{-12}	0.032
	6 (1327)	0.776 (0.093)	-4.05 (0.000)	6.7×10^{-92}	6 (223)	0.771 (0.105)	-2.37 (0.000)	5.1×10^{-8}	0.016
	12 (1801)	0.770 (0.096)	-4.81 (0.000)	8.6×10^{-137}	12 (314)	0.761 (0.096)	-3.63 (0.000)	1.4×10^{-20}	0.148
	24 (1062)	0.759 (0.100)	-6.23 (0.000)	1.5×10^{-118}	24 (180)	0.745 (0.101)	-5.60 (0.000)	4.8×10^{-34}	0.589
	48 (539)	0.740 (0.114)	-8.56 (0.000)	7.9×10^{-117}	48 (66)	0.721 (0.104)	-8.69 (0.000)	7.7×10^{-35}	0.859
**PED**	0 (1801)	0.818 (1.350)	Reference		0 (314)	0.567 (0.774)	Reference		Reference
	1 (1262)	0.534 (0.963)	-34.70 (0.071)	1.8×10^{-48}	1 (262)	0.408 (0.616)	-28.10 (0.005)	2.4×10^{-3}	0.046
	4 (1421)	0.542 (0.899)	-33.70 (0.100)	2.0×10^{-40}	4 (247)	0.376 (0.437)	-33.70 (0.002)	6.1×10^{-1}	0.003
	6 (1327)	0.517 (0.853)	-36.80 (0.099)	4.5×10^{-41}	6 (223)	0.406 (0.488)	-28.30 (0.012)	1.2×10^{-2}	0.160
	12 (1801)	0.489 (0.760)	-40.20 (0.116)	1.5×10^{-49}	12 (314)	0.391 (0.451)	-31.00 (0.104)	1.7×10^{-4}	0.084
	24 (1062)	0.454 (0.632)	-44.50 (0.032)	1.2×10^{-57}	24 (180)	0.423 (0.443)	-25.40 (0.006)	2.1×10^{-4}	0.444
	48 (539)	0.396 (0.480)	-51.60 (0.030)	1.8×10^{-67}	48 (66)	0.471 (0.510)	-16.90 (0.011)	4.6×10^{-4}	0.498

Supplementary Table S3 Mean volumes with standard deviation of segmented features in first- and second-treated eyes at baseline, one, four, six, 12, 24 and 48 months and the mean relative change (%) from baseline values with standard deviation. Segmented voxels were converted into mm^3. NSR = neurosensory retina, RPE = retinal pigment epithelium, IRF = intraretinal fluid, SRF = subretinal fluid, PED = pigment epithelium detachment, SHRM = subretinal hyperreflective material, HRF = hyperreflective foci, SD = standard deviation.
