# Supplementary material for: Quantifying Changes on OCT in Eyes Receiving Treatment for Neovascular Age-Related Macular Degeneration
Source: Ophthalmol Sci. 2024 Jun 28;4(6):100570. doi: 10.1016/j.xops.2024.100570 (PMC11367487; doi:10.1016/j.xops.2024.100570)
Supplement: Supplementary Table S4 [file mmc5.pdf]

Supplementary Table S4
Mean volumes of OCT segmented features in first-treated eyes with either Ranibizumab-only or Aflibercept-only at multiple time-points

Segmented feature	Aflibercept-only			Ranibizumab-only			
	Month (n number eyes)	Mean mm³ volume (SD)	Mean % relative change (SD)	Month (n number eyes)	Mean mm³ volume (SD)	Mean % relative change (SD)	P-value (rel. change aflibercept-only vs ranibizumab-only)
NSR	0 (1124)	9.520 (0.862)	Reference	0 (376)	9.390 (1.08)	Reference	Reference
	4 (909)	9.000 (0.792)	-5.38 (0.000)	4 (281)	8.950 (0.867)	-4.71 (0.000)	5.41x10^{-3}
	12 (1124)	8.860 (0.789)	-6.9 (0.000)	12 (376)	8.940 (0.841)	-4.77 (0.000)	**1.60x10^{-9}**
IRF	0 (1124)	0.110 (0.272)	Reference	0 (376)	0.116 (0.348)	Reference	Reference
	4 (909)	0.021 (0.099)	-81.0 (0.069)	4 (281)	0.018 (0.074)	-84.1 (0.007)	7.93x10^{-2}
	12 (1124)	0.015 (0.08)	-86.4 (0.158)	12 (376)	0.041 (0.162)	-64.8 (0.090)	**8.94x10^{-6}**
SRF	0 (1124)	0.496 (0.757)	Reference	0 (376)	0.353 (0.624)	Reference	Reference
	4 (909)	0.114 (0.327)	-77.1 (0.024)	4 (281)	0.088 (0.264)	-75.1 (0.002)	3.93x10-3
	12 (1124)	0.065 (0.238)	-87.0 (0.029)	12 (376)	0.086 (0.362)	-75.5 (0.003)	**5.10x10^{-8}**
SHRM	0 (1124)	0.369 (0.65)	Reference	0 (376)	0.314 (0.615)	Reference	Reference
	4 (909)	0.101 (0.233)	-72.7 (0.008)	4 (281)	0.099 (0.308)	-68.4 (0.002)	5.51x10^{-2}
	12 (1124)	0.085 (0.217)	-76.9 (0.022)	12 (376)	0.121 (0.33)	-61.3 (0.011)	**6.76x10^{-8}**
HRF	0 (1124)	0.003 (0.008)	Reference	0 (376)	0.003 (0.008)	Reference	Reference
	4 (909)	0.002 (0.006)	-29.7 (0.000)	4 (281)	0.002 (0.006)	-30.8 (0.001)	2.28x10^{-2}
	12 (1124)	0.001 (0.002)	-68.1 (0.001)	12 (376)	0.001 (0.004)	-57.2 (0.001)	**9.53x10^{-4}**
RPE	0 (1124)	0.815 (0.078)	Reference	0 (376)	0.790 (0.099)	Reference	Reference
	4 (909)	0.781 (0.09)	-4.18 (0.000)	4 (281)	0.764 (0.113)	-3.31 (0.000)	8.55x10^{-2}
	12 (1124)	0.773 (0.094)	-5.09 (0.000)	12 (376)	0.759 (0.109)	-3.93 (0.000)	2.28x10^{-2}
PED	0 (1124)	0.776 (1.26)	Reference	0 (376)	0.756 (1.37)	Reference	Reference
	4 (909)	0.494 (0.837)	-36.3 (0.121)	4 (281)	0.498 (0.842)	-34.1 (0.018)	5.00x10^{-1}
	12 (1124)	0.420 (0.647)	-45.9 (0.143)	12 (376)	0.532 (0.864)	-29.6 (0.024)	4.88x10^{-1}

Table S4 Mean volumes with standard deviation of segmented features in first-treated eyes with either Aflibercept-only or Ranibizumab-only and the mean relative change (%) from baseline values with standard deviation. Segmented voxels were converted into mm^3. Bolded values were significant at P<0.0002 after Bonferroni correction. NSR = neurosensory retina, RPE = retinal pigment epithelium, IRF = intraretinal fluid, SRF = subretinal fluid, PED = pigment epithelium detachment, SHRM = subretinal hyperreflective material, HRF = hyperreflective foci, N/A = Not Applicable, SD = standard deviation.
