# Supplementary material for: Quantifying Changes on OCT in Eyes Receiving Treatment for Neovascular Age-Related Macular Degeneration
Source: Ophthalmol Sci. 2024 Jun 28;4(6):100570. doi: 10.1016/j.xops.2024.100570 (PMC11367487; doi:10.1016/j.xops.2024.100570)
Supplement: Supplementary Table S5 [file mmc6.pdf]

Supplementary Table S5
Mean volumes of OCT segmented features in second-treated eyes with either Ranibizumab-only or Aflibercept-only at multiple time-points

Segmented feature	Aflibercept-only			Ranibizumab-only			
	Month (n number eyes)	Mean mm³ volume (SD)	Mean % relative change (SD)	Month (n number eyes)	Mean mm³ volume (SD)	Mean % relative change (SD)	P-value (rel. change aflibercept-only vs ranibizumab-only)
NSR	0 (230)	9.290 (0.816)	Reference	0 (46)	9.350 (0.787)	Reference	Reference
	4 (184)	8.960 (0.774)	-3.64 (0.0)	4 (34)	9.120 (0.748)	-2.41 (0.0)	0.374
	12 (230)	8.890 (0.848)	-4.32 (0.0)	12 (46)	8.910 (0.917)	-4.70 (0.0)	0.988
IRF	0 (230)	0.065 (0.159)	Reference	0 (46)	0.116 (0.252)	Reference	Reference
	4 (184)	0.009 (0.049)	-85.60 (0.028)	4 (34)	0.078 (0.293)	-33.10 (0.0)	0.237
	12 (230)	0.020 (0.141)	-68.80 (0.054)	12 (46)	0.018 (0.056)	-84.40 (0.026)	0.767
SRF	0 (230)	0.210 (0.474)	Reference	0 (46)	0.206 (0.285)	Reference	Reference
	4 (184)	0.037 (0.131)	-82.50 (0.0)	4 (34)	0.058 (0.145)	-71.70 (0.001)	0.994
	12 (230)	0.053 (0.29)	-75.0 (0.034)	12 (46)	0.071 (0.273)	-65.70 (0.012)	0.479
SHRM	0 (230)	0.132 (0.226)	Reference	0 (46)	0.145 (0.228)	Reference	Reference
	4 (184)	0.049 (0.158)	-63.00 (0.002)	4 (34)	0.059 (0.167)	-59.00 (0.008)	0.736
	12 (230)	0.048 (0.163)	-63.60 (0.002)	12 (46)	0.052 (0.147)	-64.30 (0.001)	0.198
HRF	0 (230)	0.002 (0.006)	Reference	0 (46)	0.002 (0.007)	Reference	Reference
	4 (184)	0.001 (0.003)	-32.90 (0.001)	4 (34)	0.001 (0.002)	-44.90 (0.0)	0.975
	12 (230)	0.001 (0.002)	-46.90 (0.001)	12 (46)	0.001 (0.001)	-68.40 (0.001)	0.691
RPE	0 (230)	0.793 (0.093)	Reference	0 (46)	0.773 (0.079)	Reference	Reference
	4 (184)	0.774 (0.094)	-2.38 (0.0)	4 (34)	0.762 (0.083)	-1.46 (0.0)	0.793
	12 (230)	0.764 (0.098)	-3.67 (0.0)	12 (46)	0.747 (0.095)	-3.40 (0.0)	0.762
PED	0 (230)	0.516 (0.781)	Reference	0 (46)	0.535 (0.553)	Reference	Reference
	4 (184)	0.326 (0.391)	-36.80 (0.002)	4 (34)	0.380 (0.380)	-29.00 (0.002)	0.535
	12 (230)	0.346 (0.418)	-32.90 (0.124)	12 (46)	0.452 (0.507)	-15.40 (0.002)	0.677

Table S5 Mean volumes with standard deviation of segmented features in second-treated eyes with either Aflibercept-only or Ranibizumab-only and the mean relative change (%) from baseline values with standard deviation. Segmented voxels were converted into mm^3. Bolded values were significant at P<0.0002 after Bonferroni correction. NSR = neurosensory retina, RPE = retinal pigment epithelium, IRF = intraretinal fluid, SRF = subretinal fluid, PED = pigment epithelium detachment, SHRM = subretinal hyperreflective material, HRF = hyperreflective foci, N/A = Not Applicable, SD = standard deviation.
