# Supplementary material for: Quantifying Changes on OCT in Eyes Receiving Treatment for Neovascular Age-Related Macular Degeneration
Source: Ophthalmol Sci. 2024 Jun 28;4(6):100570. doi: 10.1016/j.xops.2024.100570 (PMC11367487; doi:10.1016/j.xops.2024.100570)
Supplement: Supplementary Table S7 [file mmc7.pdf]

Supplementary Table S7

Association between retinal biomarkers at follow up and secondary exposure variables stratified by SRF baseline volume (first-treated eyes)

Characteristic		IRF volume		SRF volume		NSR volume		RPE volume		SHRM volume	
		**β (95% CI)**	***p value***	**β (95% CI)**	***p value***	**β (95% CI)**	***p value***	**β (95% CI)**	***p value***	**β (95% CI)**	***p value***
**Age**	Per decile	0.09 (-0.19, 0.37)	0.53	-0.61 (-0.82, -0.41)	**8.0 x 10^{-9}**	-0.22 (-0.27, -0.17)	**1.0 x 10^{-16}**	-0.28 (-0.33, -0.23)	**1.0 x 10^{-16}**	0.05 (-0.21, 0.31)	**1.0 x 10^{-16}**
**Sex**	Female	**Reference**		**Reference**		**Reference**		**Reference**		**Reference**	
	Male	0.17 (-0.30, 0.65)	0.48	0.00 (-0.36, 0.35)	0.99	0.06 (-0.02, 0.15)	0.15	-0.01 (-0.09, 0.08)	0.89	-0.11 (-0.55, 0.34)	0.63
**Ethnicity**	White	**Reference**		**Reference**		**Reference**		**Reference**		**Reference**	
	Asian	-0.67 (-1.19, -0.15)	1.2 x 10^{-2}	-0.30 (-0.68, 0.09)	0.14	-0.19 (-0.28, -0.09)	**8.8 x 10^{-5}**	-0.04 (-0.13, 0.05)	0.41	0.30 (-0.19, 0.78)	0.24
	Black	1.87 (0.17, 3.57)	3.1 x 10-2	0.07 (-1.18, 1.33)	0.91	-0.46 (-0.77, -0.16)	3.0 x 10^{-3}	0.33 (0.03, 0.64)	3.2 x 10^{-2}	0.65 (-0.93, 2.24)	0.42
	Other	-0.07 (-0.87, 0.73)	0.87	-0.35 (-0.95, 0.24)	0.24	-0.20 (-0.34, -0.05)	7.0 x 10^{-3}	0.00 (-0.14, 0.14)	1	0.70 (-0.05, 1.44)	0.07
**Time**	Per month	-0.30 (-0.35, -0.25)	**1.0 x 10^{-16}**	-0.31 (-0.35, -0.27)	**1.0 x 10^{-16}**	-0.04 (-0.04, -0.03)	**1.0 x 10^{-16}**	-0.02 (-0.02, -0.01)	**6.6 x 10^{-11}**	-0.27 (-0.31, -0.23)	**1.0 x 10^{-16}**
**Visual acuity**	Per letter	-0.08 (-0.09, -0.06)	**1.0 x 10^{-16}**	-0.02 (-0.03, 0.00)	9.0 x 10^{-3}	0.00 (0.00, 0.00)	0.12	0.01 (0.01, 0.02)	**1.0 x 10^{-16}**	-0.08 (-0.09, -0.06)	**1.0 x 10^{-16}**
**Baseline SRF volume**	Low	**Reference**		**Reference**		**Reference**		**Reference**		**Reference**	
	High	0.54 (-0.04, 1.11)	0.07	3.85 (3.40, 4.29)	**1.0 x 10^{-16}**	0.25 (0.16, 0.34)	**8.7 x 10^{-8}**	0.27 (0.18, 0.36)	**5.0 x 10^{-9}**	2.29 (1.78, 2.80)	**1.0 x 10^{-16}**
**Time: high SRF**	Interaction	0.02 (-0.05, 0.09)	0.61	-0.10 (-0.16, -0.04)	2.0 x 10^{-3}	-0.03 (-0.04, -0.02)	**4.4 x 10^{-15}**	-0.03 (-0.04, -0.02)	**1.0 x 10^{-16}**	0.00 (-0.06, 0.06)	0.98

Supplementary Table S7: Association between retinal biomarkers at follow up and secondary exposure variables stratified by SRF baseline volume. Note that the interaction effect indicates rate of biomarker change. Bolded values were significant at P<0.00026 after Bonferroni correction. NSR = neurosensory retina, RPE = retinal pigment epithelium, IRF = intraretinal fluid, SRF = subretinal fluid, PED = pigment epithelium detachment, SHRM = subretinal hyperreflective material, HRF = hyperreflective foci, CI= Confidence interval.
