# Supplementary material for: Quantifying Changes on OCT in Eyes Receiving Treatment for Neovascular Age-Related Macular Degeneration
Source: Ophthalmol Sci. 2024 Jun 28;4(6):100570. doi: 10.1016/j.xops.2024.100570 (PMC11367487; doi:10.1016/j.xops.2024.100570)
Supplement: Supplementary Table S8 [file mmc8.pdf]

Supplementary Table S8

Association between retinal biomarkers at follow up and secondary exposure variables stratified by IRF baseline volume (first-treated eyes)

**Characteristic**		**IRF volume**		**SRF volume**		**NSR volume**		**RPE volume**		**SHRM volume**	
		**β (95% CI)**	***p value***	**β (95% CI)**	***p value***	**β (95% CI)**	***p value***	**β (95% CI)**	***p value***	**β (95% CI)**	***p value***
**Age**	Per decile	-0.26 (-0.51, -0.02)	0.04	-0.83 (-1.06, -0.61)	**1.0×10^{-12}**	-0.24 (-0.29, -0.19)	**1.0×10^{-16}**	-0.29 (-0.33, -0.24)	**1.0×10^{-16}**	-0.19 (-0.45, 0.08)	0.17
**Sex**	Female	**Reference**		**Reference**		**Reference**		**Reference**		**Reference**	
	Male	0.11 (-0.31 ,0.54)	0.6	0.06 (-0.45, 0.34)	0.78	0.05 (-0.03, -0.14)	0.21	-0.01 (-0.10, 0.07)	0.81	-0.15 (-0.61, 0.31)	0.52
**Ethnicity**	White	**Reference**		**Reference**		**Reference**		**Reference**		**Reference**	
	Asian	-0.78 (-1.24, -0.31)	1.0×10^{-3}	0.20 (-0.63, 0.23)	0.35	-0.19 (-0.28, -0.10)	**8.0×10^{-5}**	-0.03 (-0.13, 0.06)	0.47	0.32 (-0.18, 0.82)	0.21
	Black	1.60 (0.06, 3.05)	0.04	0.32 (-1.07, 1.71)	0.65	-0.48 (-0.79, -0.17)	2.0 x 10-3	0.34 (0.03, 0.64)	0.03	0.73 (-0.90, 2.35)	0.38
	Other	-0.14 (-0.85, 0.57)	0.7	-0.36 (-1.02, 0.29)	0.28	-0.20 (-0.34, -0.05)	8.0×10^{-3}	0.00 (-0.14, 0.15)	0.98	0.66 (-0.10, 1.43)	0.09
**Time**	Per month	-0.10 (-0.15, -0.05)	**8.0×10^{-5}**	-0.28 (-0.32, -0.23)	**1.0×10^{-16}**	-0.03 (-0.03, -0.02)	**1.0×10^{-16}**	-0.02 (-0.03, -0.02)	**1.0×10^{-16}**	-0.24 (-0.29, -0.20)	**1.0×10^{-16}**
**Visual acuity**	Per letter	-0.04 (-0.05, -0.03)	**3.3×10^{-8}**	-0.03 (-0.04, -0.02)	**2.6×10^{-6}**	0.00 (0.00, 0.01)	0.02	0.01 (0.01, 0.01)	**9.1×10^{-16}**	-0.08 (-0.09, -0.06)	**1.0×10^{-16}**
**Baseline IRF volume**	Low	**Reference**		**Reference**		**Reference**		**Reference**		**Reference**	
	High	6.34 (5.78, 6.87)	**1.0×10^{-16}**	0.02 (-0.46, 0.50)	0.95	0.41 (0.32, 0.50)	**1.0×10^{-16}**	0.01 (-0.08, 0.10)	0.86	1.12 (0.59, 1.66)	**4.1×10^{-5}**
**Time: high IRF**	Interaction	-0.37 (-0.44, -0.30)	**1.0 x 10-16**	-0.17 (-0.23, -0.11)	**7.4×10^{-8}**	-0.05 (-0.06, -0.04)	**1.0×10^{-16}**	-0.02 (-0.02, -0.01)	**1.2×10^{-5}**	-0.05 (-0.11, 0.01)	0.11

Supplementary Table S8: Association between retinal biomarkers at follow up and secondary exposure variables stratified by IRF baseline volume. Note that the interaction effect indicates rate of biomarker change. Bolded values were significant at P<0.00026 after Bonferroni correction. NSR = neurosensory retina, RPE = retinal pigment epithelium, IRF = intraretinal fluid, SRF = subretinal fluid, PED = pigment epithelium detachment, SHRM = subretinal hyperreflective material, HRF = hyperreflective foci, CI= Confidence interval.
